# Association of Mid- to Late-Life Blood Pressure Patterns With Risk of Subsequent Coronary Heart Disease and Death

**DOI:** 10.3389/fcvm.2021.632514

**Published:** 2021-02-15

**Authors:** Menghui Liu, Shaozhao Zhang, Xiaohong Chen, Xiangbin Zhong, Zhenyu Xiong, Daya Yang, Yifen Lin, Yiquan Huang, Yuqi Li, Lichun Wang, Xiaodong Zhuang, Xinxue Liao

**Affiliations:** ^1^Department of Cardiology, The First Affiliated Hospital of Sun Yat-sen University, Guangzhou, China; ^2^NHC Key Laboratory of Assisted Circulation (Sun Yat-sen University), Guangzhou, China; ^3^Department of Otorhinolaryngology, The Third Affiliated Hospital of Sun Yat-sen University, Guangzhou, China

**Keywords:** blood pressure, midlife hypertension, all-cause mortality, coronary heart disease, old age

## Abstract

**Background:** The elevated blood pressure (BP) at midlife or late-life is associated with cardiovascular disease and death. However, there is limited research on the association between the BP patterns from middle to old age and incident coronary heart disease (CHD) and death.

**Methods:** A cohort of the Atherosclerosis Risk in Communities (ARIC) Study enrolled 9,829 participants who attended five in-person visits from 1987 to 2013. We determined the association of mid- to late-life BP patterns with incident CHD and all-cause mortality using multivariable-adjusted Cox proportional hazards models.

**Results:** During a median of 16.7 years of follow-up, 3,134 deaths and 1,060 CHD events occurred. Compared with participants with midlife normotension, the adjusted hazard ratio for all-cause mortality and CHD was 1.14 (95% CI, 1.04–1.25) and 1.28 (95% CI, 1.10–1.50) in those with midlife hypertension, respectively. In further analyses, compared with a pattern of sustained normotension from mid- to late-life, there was no significant difference for the risk of incident death (HR, 1.15; 95% CI, 0.96–1.37) and CHD (HR, 1.33; 95% CI, 0.99–1.80) in participants with a pattern of midlife normotension and late-life hypertension with effective BP control. A higher risks of death and CHD were found in those with pattern of mid- to late-life hypertension with effective BP control (all-cause mortality: HR, 1.24; 95% CI, 1.08–1.43; CHD: HR, 1.65; 95% CI 1.30–2.09), pattern of midlife normotension and late-life hypertension with poor BP control (all-cause mortality: HR, 1.27; 95% CI, 1.12–1.44; CHD: HR, 1.53; 95% CI, 1.23–1.92), and pattern of mid- to late-life hypertension with poor BP control (all-cause mortality: HR, 1.49; 95% CI, 1.30–1.71; CHD: HR, 1.87; 95% CI, 1.48–2.37).

**Conclusions:** The current findings underscore that the management of elderly hypertensive patients should not merely focus on the current BP status, but the middle-aged BP status. To achieve optimal reductions in the risk of CHD and death, it may be necessary to prevent, diagnose, and manage of hypertension throughout middle age.

## Introduction

The prevalence of hypertension increases progressively with age, and so the majority of elderly (age ≥60 years) are hypertensive (1). Late-life hypertension is a strong cardiovascular risk factor and a major contributor to premature disability and death (2–4). Clinical trials have shown that treatment of hypertension substantially reduces the risks of cardiovascular disease (CVD) and mortality in elderly population (5, 6). However, the residual risk of CVD and death in those treated for hypertension is always present (7). Of note, long-term changes of blood pressure (BP) (8, 9) and antecedent BP levels (10–13) may further influence the risk of CVD and death. Prior studies have reported that the health status (14, 15) and the higher risks of CVD and death (10–13) might be associated with elevated midlife BP in older adults, independent of late life BP. Thus, midlife BP status might be associated with the residual risk of CVD in older adults. To optimize clinical and public health strategies toward minimizing the burden of CVD, a much-needed first step is to describe the association among midlife BP, late-life BP, and the CVD risk. However, little is known regarding the relationship between the mid- to late-life BP patterns and subsequent CVD and death in older adults.

Therefore, the aim of our study was to identify the association of midlife and late-life BP patterns with risk of subsequent coronary heart disease (CHD) and death using data from the Atherosclerosis Risk in Communities (ARIC) study (16).

## Materials and Methods

The data and materials from the present study will not be made available by the authors for the purpose of reproducing the results because of human subjects' restrictions. However, interested investigators can contact the ARIC Study Coordinating Center to request overall access to ARIC Study data.

### Study Design and Study Population

The ARIC study (16) was a prospective, epidemiological cohort to evaluate the risk factors for CVD, and initially enrolled 15,792 participants (aged 45–65 years) from four US population centers between 1987 and 1989: Washington County, Maryland; Forsyth County, North Carolina; northwestern suburbs of Minneapolis, Minnesota; and Jackson, Mississippi. After the baseline examination, three subsequent study visits were conducted in person every 3 years: visit 2 (1990–1992), visit 3 (1993–1995), and visit 4 (1996–1998). Fifteen years later, a fifth visit occurred between 2011 and 2013. These participants have been followed continuously for CVD events through annual or semiannual telephone interviews and active surveillance of ARIC study community hospitals.

**Figure 1:**
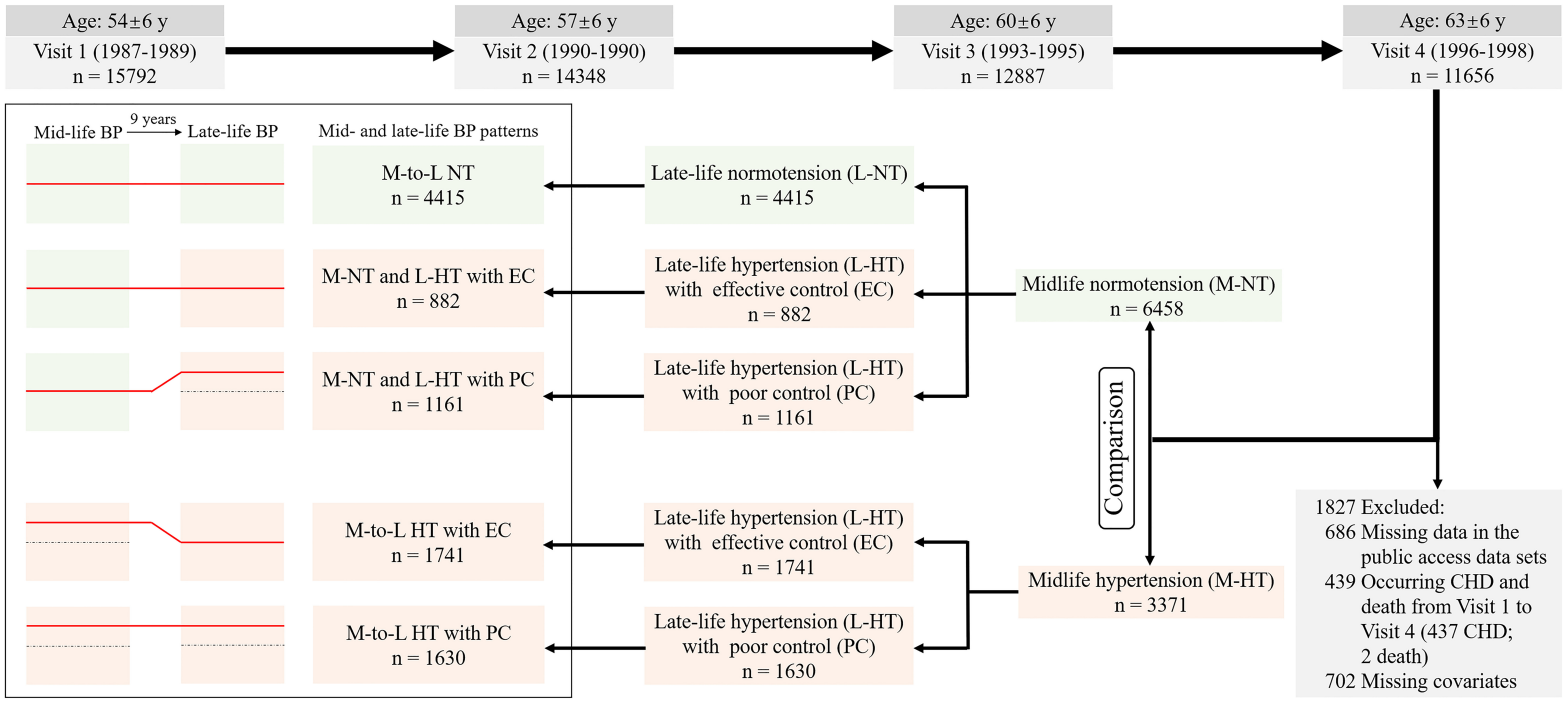
Flow diagram for the cohort selection and the classification of mid- and late-life blood pressure patterns. Salmon pink represent hypertension; Light green represent normotension; Solid red lines represent the blood pressure (BP) pattern from middle to old age. Effective BP control (EC) was defined at visit 4 as 140 mm Hg > SBP ≥ 90 mm Hg and 90 mm Hg > DBP ≥ 60 mm Hg in patients with hypertension, irrespective of current antihypertensive medication used, otherwise it will be defined as poor BP control (PC). M-to-L NT, mid- to late-life normotension; M-NT and L-HT with EC, midlife normotension and late-life hypertension with effective control; M-to-L HT with EC, mid- to late-life hypertension with effective control; M-NT and L-HT with PC, midlife normotension and late-life hypertension with poor control; M-to-L HT with PC, mid- to late-life hypertension with poor control.

For this analysis, we included 9,829 participants who attended visit 4, excluding those missing data in the public access data sets (*n* = 686); occurring the CHD (*n* = 437) or death event (*n* = 2) from visit 1 to visit 4; missing BP data at visit 1 or visit 4 (*n* = 5), or information on covariates (*n* = 697) (Figure 1). All participants provided informed consent, and the institutional review boards at all participating institutions approved the ARIC study protocol.

### Assessment of Mid- to Late-Life BP Patterns

Technicians measured three seated BP readings after a 5-min rest using a random zero sphygmomanometer. The average of the last two measures was used for analysis at visit 1 (midlife) and visit 4 (late-life). Midlife/middle age was defined as the people aged 45–60, which was described by visit 1 (54 ± 6 years) in this study, and late-life/old age was defined as the people aged 60–75, which was described by visit 4 (63 ± 6 years) in this study. Hypertension was defined as systolic BP (SBP) above 140 mm Hg or diastolic BP (DBP) above 90 mm Hg or hypertension previously diagnosed or taking any of the medications during the past 2 weeks for hypertension, even if the individual's BP was in the normal range (4). Based on the BP treatment target (<140/90 mmHg) of current guideline (4) and the definition of hypotension (<90/60 mmHg) (17), effective BP control (EC) was defined at visit 4 as 140 mm Hg > SBP ≥ 90 mm Hg and 90 mm Hg > DBP ≥ 60 mm Hg in patients with hypertension, irrespective of current antihypertensive medication used, otherwise it will be defined as poor BP control (PC). The diagnoses (normotension, hypertension) at midlife and late-life and the BP levels (effective control; poor control) at old age were used to describe the longitudinal BP patterns from middle to old age. Midlife hypertension (M-HT) was defined as meeting hypertension criteria at visits 1 (*n* = 3,371), and the remaining persons not meeting these criteria were classified as midlife normotension (M-NT) (*n* = 6,458). These participants were further divided into one of the five categories of mid- to late-life BP patterns according to the above-mentioned criteria (Figure 1). The reference group: Mid- to late-life normotension (M-to-L NT) (*n* = 4,415); Midlife normotension and late-life hypertension with effective control (M-NT and L-HT with EC) (*n* = 882); Midlife normotension and late-life hypertension with poor control (M-NT and L-HT with PC) (*n* = 1,161); Mid- to late-life hypertension with effective control (M-to-L HT with EC) (*n* = 1,741); Mid- to late-life hypertension with poor control (M-to-L HT with PC) (*n* = 1,630).

### Outcomes Assessed

The ascertainment of deaths and CHD events in ARIC have been described previously (18, 19). All-cause mortality was defined as death from any cause, and was ascertained through the review of hospital discharge records and death certificates, supplemented by informant interviews or physician questionnaires for out-of-hospital deaths (18). The CHD events were adjudicated by an ARIC end points committee and included fatal CHD, definite or probable MI, and silent MI (as determined by ARIC examination ECGs) (19).

### Statistical Analyses

Baseline data at visit 4 are presented as mean [standard deviation (SD)] or number (%). The characteristics of participants were compared between the midlife hypertension and midlife normotension groups, and among five groups of mid- to late-life BP pattern using the one-way ANOVA test, the Pearson χ^2^-test, or the Kruskal–Wallis test, as appropriate.

Kaplan–Meier survival function curves were calculated for the all-cause mortality and CHD events across the midlife hypertension and midlife normotension groups. The associations between the midlife hypertension and the risk of death and CHD were assessed using multivariable-adjusted Cox proportional hazards models. The demographic variables or the variables that might be associated with death in the results of univariate analysis (Supplementary Table 1) or in previous literature reported were included in the model. Separate models were constructed for death and CHD with inclusion of the following covariates: Model 1 included age, sex, race at visit 4; Model 2 included variables in model 1 plus education level, smoking status, drinking status, body mass index (BMI), total cholesterol, high-density lipoprotein cholesterol (HDL-C), low-density lipoprotein cholesterol (LDL-C), estimated glomerular filtration rate (eGFR), prevalent diabetes mellitus, coronary heart disease, stroke and heart failure, use of aspirin and statin at visit 4; Model 3 included variables in model 2 and SBP, DBP, use of antihypertensive drugs at visit 4. Then, we further constructed multivariable-adjusted Cox proportional hazards models to estimate the hazard ratios (HR) and 95% confidence intervals (CI) for incident death and CHD associated with mid- to late-life BP patterns. The M-to-L NT group was used as the referent to which each of the groups was compared on the all-cause mortality and CHD events. The SBP and DBP were excluded in the inclusion of covariates in multivariable-adjusted Cox proportional hazards models in that the classification of mid- to late-life BP patterns were partly based on the SBP, DBP at old age.

For subgroup analyses of the all-cause mortality and CHD events, an interaction term between variables of interest and mid- to late-life BP patterns was individually added to the above multivariable-adjusted Cox proportional hazards model, and the *P*-values and CIs for these associations were estimated.

A *post-hoc* analysis was conducted in those without CHD, MI, stroke, or heart failure (HF). The participants with CHD, MI, stroke, or HF at visit 4 were excluded (*n* = 1,023). The remaining participants (*n* = 8,806) were used to assess the association between the mid- to late-life BP patterns and the risk of death and CHD. Previous studies pointed that hypotension defined as SBP < 90 mm Hg or DBP < 60 mm Hg might also be associated with an increased risk of CVD or death, especially in older adults (20, 21). We thus excluded the participants who were defined as hypotension at visit 1 (*n* = 829) and visit 4 (*n* = 885), and performed a sensitivity analysis in participants without hypotension at visit 1 and 4. Moreover, to more accurately assess the BP status of middle and old age, a sensitivity analysis was conducted after excluding the participants who were more than 60 years at visit 1 (*n* = 2,081) or <60 years at visit 4 (*n* = 3,304). Additionally, a *post-hoc* analysis was further performed when midlife hypertension was also classified into EC and PC groups. The multivariable-adjusted Cox proportional hazards models were used in these analyses.

Furthermore, the E-value also used to assess the robustness of the identified association between mid- to late-life BP patterns and the risk of death and CHD to potential unmeasured confounders (These detailed explanations and methods in the online-only Data Supplement) (22).

A significance level of <0.05 for two-sided comparisons was considered statistically significant, and 95% CIs were reported where applicable. Because of the potential for type 1 error due to multiple comparisons, findings for analyses of secondary end points should be interpreted as exploratory. Analyses were conducted using the statistical program Stata Version 14 (StataCorp) and the R language (version 3.5.0.12).

## Results

### Association of Midlife Hypertension With Incident Death and Coronary Heart Disease (CHD)

The current study included 9,829 participants from the ARIC study, who were grouped into midlife hypertension (*n* = 3,371) vs. midlife normotension (*n* = 6,458) with an average of 63 years old at visit 4 (average 54 years old at visit 1), 4,288 (43.6 %) men, and 7,826 (79.6 %) white races. The participant characteristics at visit 4 stratified by the status of midlife BP are shown in Supplementary Table 2. Compared with the midlife normotension group, the participants with midlife hypertension were older, more commonly women, more likely black, higher BMI, lower eGFR, more previously diagnosed as diabetes, CHD, stroke, heart failure, and had fewer drinker, lower aspirin, and statin user.

**Figure 2:**
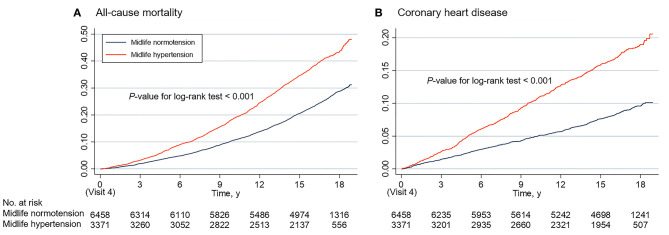
Cumulative incidence estimates (Kaplan–Meier) for the all-cause mortality **(A)** and coronary heart disease **(B)** between midlife normotension and midlife hypertension groups.

During a median follow-up of 16.7 years, 3,134 incident deaths and 1,060 incident CHD occurred in included participants. The higher risks of death and CHD in midlife hypertension group are shown in Kaplan–Meier survival function curves (Figure 2). In multivariable adjusted Cox proportional hazards model, the higher risks of all-cause mortality (HR 1.14; 95% CI 1.04–1.25) and CHD (HR 1.28; 95% CI 1.10–1.50) were also found in the participants with midlife hypertension compared with the midlife normotension group (Supplementary Table 3).

### Association of Mid- and Late-Life BP Patterns With Incident Death and Coronary Heart Disease (CHD)

The included participants with or without midlife hypertension were further classified as the five mid- to late-life BP patterns according to the BP status (normotension; hypertension) and levels (effective control; poor control) at old age. The characteristics of these participants at visit 4 are shown in Table 1. The significant differences were found in age, gender, race, BMI, eGFR, history of diabetes, CHD, stroke and HF, use of aspirin, and statin among the different mid- and late-life BP patterns. Therefore, the multivariable adjusted Cox proportional hazards model was used to assess the association between mid- and late-life BP patterns with outcomes.

**Table 1:** Participant characteristics at visit 4 stratified by mid- to late-life blood pressure (BP) patterns.

**Characteristic, *n* (%) or mean (SD)**	**M-to-L NT**	**M-NT and L-HT with EC**	**M-NT and L-HT with PC**	**M-to-L HT with EC**	**M-to-L HT with PC**	***P*-Value**
No.	4,415	882	1,161	1,741	1,630	
Age, y	61.8 ± 5.5	62.3 ± 5.5	63.9 ± 5.7	62.9 ± 5.6	65.1 ± 5.5	<0.001
Sex, No. (%)						<0.001
Men	1,979 (44.8)	441 (50.0)	495 (42.6)	736 (42.3)	637 (39.1)	
Women	2,436 (55.2)	441 (50.0)	666 (57.4)	1,005 (57.7)	993 (60.9)	
Race, No. (%)						<0.001
Black	528 (12.0)	189 (21.4)	209 (18.0)	541 (31.1)	536 (32.9)	
White	3,887 (88.0)	693 (78.6)	952 (82.0)	1,200 (68.9)	1,094 (67.1)	
BMI, kg/m^2^	27.3 ± 4.7	29.7 ± 5.5	28.7 ± 5.6	30.4 ± 6.1	30.2 ± 6.1	<0.001
Systolic BP, mm Hg	117.4 ± 11.8	121.1 ± 10.6	144.4 ± 17.5	123.8 ± 10.4	149.1 ± 19.7	<0.001
Diastolic BP, mm Hg	68.1 ± 8.5	70.7 ± 6.5	74.5 ± 12.5	71.8 ± 7.4	74.9 ± 14.1	<0.001
Total cholesterol, mmol/L	5.2 ± 0.9	5.1 ± 0.9	5.2 ± 0.9	5.1 ± 1.0	5.2 ± 1.0	0.004
HDL-C, mmol/L	1.3 ± 0.4	1.3 ± 0.4	1.3 ± 0.4	1.3 ± 0.4	1.3 ± 0.4	<0.001
LDL-C, mmol/L	3.2 ± 0.9	3.1 ± 0.8	3.2 ± 0.9	3.1 ± 0.9	3.2 ± 0.9	0.007
eGFR, mL/min/1.73 m^2^	88.0 ± 13.1	85.5 ± 15.8	85.5 ± 15.8	85.1 ± 17.7	82.9 ± 19.2	<0.001
History of diabetes, No. (%)	376 (8.5)	163 (18.5)	173 (14.9)	379 (21.8)	447 (27.4)	<0.001
History of CHD, No. (%)	86 (1.9)	63 (7.1)	49 (4.2)	135 (7.8)	143 (8.8)	<0.001
History of stroke, No. (%)	31 (0.7)	17 (1.9)	20 (1.7)	68 (3.9)	80 (4.9)	<0.001
History of HF, No. (%)	15 (0.3)	19 (2.2)	22 (1.9)	210 (12.1)	197 (12.1)	<0.001
Education level, No. (%)						<0.001
Basic or 0 y	596 (13.5)	161 (18.3)	200 (17.2)	373 (21.4)	453 (27.8)	
Intermediate	1,901 (43.1)	371 (42.0)	508 (43.8)	732 (42.1)	657 (40.3)	
Advanced	1,918 (43.4)	350 (39.7)	453 (39.0)	636 (36.5)	520 (31.9)	
Smoking, No. (%)						<0.001
Current smoker	730 (16.5)	124 (14.1)	181 (15.6)	213 (12.2)	211 (12.9)	
Former smoker	1,866 (42.3)	412 (46.7)	528 (45.5)	768 (44.1)	689 (42.3)	
Never smoker	1,819 (41.2)	346 (39.2)	452 (38.9)	760 (43.7)	730 (44.8)	
Drinking, No. (%)						<0.001
Current drinker	2,503 (56.7)	432 (49.0)	612 (52.7)	786 (45.2)	628 (38.6)	
Former drinker	1,174 (26.6)	283 (32.1)	313 (27.0)	556 (31.9)	550 (33.7)	
Never drinker	738 (16.7)	167 (18.9)	236 (20.3)	399 (22.9)	452 (27.7)	
Aspirin, No. (%)	2,201 (49.9)	560 (63.5)	679 (58.5)	1,070 (61.5)	989 (60.7)	<0.001
Statin, No. (%)	277 (6.3)	147 (16.7)	125 (10.8)	251 (14.4)	236 (14.5)	<0.001
Antihypertensive, No. (%)	0 (0)	882 (100)	473 (40.7)	1,415 (81.3)	1,319 (80.9)	<0.001
**Characteristics at midlife (Visit 1)**						
Age, y	52.8 ± 5.5	53.4 ± 5.5	54.9 ± 5.7	54.0 ± 5.6	56.2 ± 5.5	<0.001
Systolic BP, mm Hg	110.1 ± 11.2	118.8 ± 11.4	121.3 ± 10.9	125.0 ± 18.3	135.9 ± 19.5	<0.001
Diastolic BP, mm Hg	68.6 ± 8.2	74.1 ± 8.0	72.7 ± 8.7	77.9 ± 11.5	79.1 ± 11.7	<0.001

*Data presented as mean (SD) or percentage. BP, blood pressure; M-to-L NT, mid- to late-life normotension; M-NT and L-HT with EC, midlife normotension and late-life hypertension with effective control; M-NT and L-HT with PC, midlife normotension and late-life hypertension with poor control; M-to-L HT with EC, mid- to late-life hypertension with effective control; M-to-L HT with PC, mid- to late-life hypertension with poor control; BMI, indicates body mass index; HDL-C, high-density lipoprotein cholesterol; LDL-C, low-density lipoprotein cholesterol; eGFR, estimated glomerular filtration rate; CHD: coronary heart disease; HF, heart failure*.

As shown in the adjusted Cox proportional hazards model for traditional risk factors in Table 2, there were different risks with or without midlife hypertension in older adults with late-life hypertension and effective BP control. Compared with the M-to-L NT group, the M-to-L HT with EC group were significantly associated with increased risks of all-cause mortality (HR 1.24; 95% CI 1.08–1.43) and CHD (HR 1.65; 95% CI 1.30–2.09), but not in M-NT and L-HT with EC group (all-cause mortality: HR 1.15; 95% CI 0.96–1.37; CHD: HR 1.33; 95% CI 0.99–1.80) (Table 2).

**Table 2:** Hazard ratios from the Cox models for all-cause mortality and coronary heart disease among mid- to late-life BP models.

**Outcome**	**No./Total No. (%)**	**Model 1**		**Model 2**		**Model 3**	
		**HR (95% CI)**	***P*-Value**	**HR (95% CI)**	***P*-Value**	**HR (95% CI)**	***P*-Value**
**All-cause mortality**							
M-to-L NT	1,045/4,415 (23.7)	1.00 (Reference)	—	1.00 (Reference)	—	1.00 (Reference)	—
**L-HT with EC**							
M-NT and L-HT with EC	264/882 (29.9)	1.22 (1.07–1.40)	0.004	1.10 (0.96–1.26)	0.193	1.15 (0.96–1.37)	0.124
M-to-L HT with EC	618/1,741 (35.5)	1.44 (1.30–1.59)	<0.001	1.20 (1.07–1.33)	0.001	1.24 (1.08–1.43)	0.002
**L-HT with PC**							
M-NT and L-HT with PC	411/1,161 (35.4)	1.35 (1.20–1.51)	<0.001	1.24 (1.10–1.39)	<0.001	1.27 (1.12–1.44)	<0.001
M-to-L HT with PC	796/1,630 (48.8)	1.78 (1.62–1.96)	<0.001	1.44 (1.30–1.59)	<0.001	1.49 (1.30–1.71)	<0.001
**Coronary heart disease**							
M-to-L NT	292/4,415 (6.6)	1.00 (Reference)	—	1.00 (Reference)	—	1.00 (Reference)	—
**L-HT with EC**							
M-NT and L-HT with EC	99/882 (11.2)	1.68 (1.34–2.11)	<0.001	1.34 (1.06–1.69)	0.013	1.33 (0.99–1.80)	0.059
M-to-L HT with EC	244/1,741 (14.0)	2.21 (1.86–2.62)	<0.001	1.66 (1.38–1.99)	<0.001	1.65 (1.30–2.09)	<0.001
**L-HT with PC**							
M-NT and L-HT with PC	141/1,161 (12.1)	1.81 (1.48–2.22)	<0.001	1.54 (1.25–1.89)	<0.001	1.53 (1.23–1.92)	<0.001
M-to-L HT with PC	284/1,630 (17.4)	2.68 (2.26–3.18)	<0.001	1.88 (1.57–2.26)	<0.001	1.87 (1.48–2.37)	<0.001

*Model 1: adjusted for age, sex, race at Visit 4*.

*Model 2: adjusted for model 1 + education level, smoking status, drinking status, BMI, total cholesterol, HDL-C, LDL-C, eGFR, prevalent diabetes mellitus, coronary heart disease, stroke and heart failure, use of aspirin and statin at Visit 4*.

*Model 3: adjusted for model 2 + use of antihypertensive drugs at Visit 4*.

*M-to-L NT, mid- to late-life normotension; M-NT and L-HT with EC, midlife normotension and late-life hypertension with effective control; M-to-L HT with EC, mid- to late-life hypertension with effective control; M-NT and L-HT with PC, midlife normotension and late-life hypertension with poor control; M-to-L HT with PC, mid- to late-life hypertension with poor control; BP, blood pressure; BMI, body mass index; HDL-C, high-density lipoprotein cholesterol; LDL-C, low-density lipoprotein cholesterol; eGFR, estimated glomerular filtration rate*.

Similarly, in older adults with late-life hypertension and poor BP control, there was a much higher risk in those who were diagnosed with hypertension at the middle age. Compared with the M-to-L NT group, the M-NT, and L-HT with PC group was just associated with a 27% higher risk of all-cause mortality (HR 1.27; 95% CI 1.12–1.44) and a 53% higher risk of CHD (HR 1.53; 95% CI 1.23–1.92). However, the M-to-L HT with PC group was associated with a 49% higher risk of all-cause mortality (HR 1.49; 95% CI 1.30–1.71) and an 87% higher risk of CHD (HR 1.87; 95% CI 1.48–2.37) (Table 2).

Furthermore, we conducted the subgroup analyses for key variables and tested the interaction. There were different risks of death and CHD in subgroup of sex, race, BMI, LDL-C level, and previous diabetes history, but interaction testing revealed no heterogeneity in the association of mid- and late-life BP patterns with incident death and CHD based on these variables. In the subgroup analyses of age, the similar risks of CHD were found in the subgroup of age <65 or ≥65 years without the interaction, but the much higher risks of death in subgroup of age <65 years were found in M-to-L HT with PC group compared with the M-to-L NT group (HR 2.12; 95% CI 1.71–2.64), with significant interaction (*P* for interaction <0.001) (Supplementary Figures 1, 2).

### Sensitivity Analyses

The sensitivity analyses were conducted in those without CHD, MI, stroke, or HF (*n* = 8,806) and those without hypotension at visit 1 and 4 (*n* = 8,115). The results of these analyses also found that the midlife hypertension was associated with the higher risks of all-cause mortality and CHD (Supplementary Tables 4, 5). Further analyses were performed in 5 mid- and late-life BP patterns, and revealed the similar results to the primary analyses (Supplementary Tables 6, 7). In sensitivity analysis of participants with the age < 60 years in visit 1 (Mid-life) and the age ≥ 60 years in visit 4 (Late-life), we also found the similar results to the primary analyses (Supplementary Table 8).

When midlife hypertension was also classified into EC and PC groups, we found that the midlife hypertension with poor BP control was significantly associated with the higher risks of CHD and death in elderly hypertensive patients, whether the BP was effectively controlled in old age or not (Supplementary Table 9). Among the participants with midlife hypertension with effective BP control, the poor BP control at old age was also associated with the higher risks of CHD and death. Of note, the higher risk of death was not found in participants with a pattern of midlife hypertension with effective BP control and late-life hypertension with effective BP control (Supplementary Table 9). These results further suggested that the impact of midlife hypertension on prognosis for older adults, and the participants with midlife hypertension could benefit from the effective BP control in middle age.

The E-values for the all-cause mortality and CHD events were evaluated and compared with the HRs of known cardiovascular risk factors for these outcomes. The results revealed that using an unmeasured confounder to explain the observed association between mid- and late-life BP patterns and these outcomes was unlikely (Supplementary Table 10).

## Discussion

In present prospective cohort study, several important findings were observed. First, the midlife hypertension was associated with the higher risks of all-cause mortality and CHD events in older adults. Second, compared with a pattern of sustained normotension from mid- to late-life, among elderly hypertensive patients with effective BP control, those with midlife hypertension was associated with the higher risks of all-cause mortality and CHD events. Of note, the risks of all-cause mortality and CHD events were not found in those with midlife normotension. Third, among elderly hypertensive patients with poor BP control, those with midlife hypertension was associated with the much higher risks of all-cause mortality and CHD events than those without midlife hypertension. Our findings suggested that the elderly hypertensive patient with midlife hypertension may be a distinctive individual at higher risk of death and CHD. Therefore, the management of elderly hypertensive patients should not merely focus on the current BP status, but the middle-aged BP status.

Previous studies have noted the effect of antecedent BP level on the risks of CVD mortality and stroke several decades later (10–13, 23–25). Four decades ago, a report based on the Hiroshima and Nagasaki Adult Health Study identified that, in addition to current SBP, the SBP 2–4 years before baseline did predict the future risk of stroke in middle-aged Japanese adults. However, this study did not assess the effect of remote antecedent BP (23). A prospective cohort from Framingham Heart Study was the earlier study to explore the association between elevated midlife BP and stroke risk in 5,197 stroke-free community population (11). The results suggested that midlife BP levels continue to affect the future risk of stroke not only over a short span, such as 5 years, but over more prolonged periods, up to 30 years (11), with similar findings in the analysis of data from seven diverse US cohort studies (10). Recently, Gill et al. (13) conducted univariable and multivariable mendelian randomization to investigate the effect of genetically predicted mean arterial pressure (MAP) at age ≤ 55 years on coronary artery disease (CAD) risk using genetic association estimates from the UK Biobank and CARDIoGRAMplusC4D consortium, and the results supported an effect of midlife BP on late-life CAD risk that is independent of late-life BP (13). These studies were more focused on the impact of midlife BP level, but not on the middle-aged BP status. Our study extended those findings and demonstrated the significance of middle-aged BP status in older adults. The elderly hypertensive patients experienced midlife hypertension placing them at higher risks of death and CHD. Therefore, taking middle-aged BP status into account might provide more accurate estimates for the risks of death and CHD events, and represent a step forward in developing the individualized risk prediction model.

The 2017 ACC/AHA guideline (26) and 2018 ESC/ESH guideline (4) always recommend that the BP management for the elderly should be based on current BP levels. However, in those patients who have received the guideline-recommended treatment for hypertension, the residual risk of CVD and death is always present (7). In current study, among elderly people with hypertension and effective BP control, the higher risks of all-cause mortality and CHD were found in the participants with midlife hypertension, but not in participants with midlife normotension. The finding suggested that midlife hypertension might partly explain the residual risk of CVD and death. Our work generated the evidence supporting that the management of elderly hypertensive patients should not merely focus on the current BP status, but the middle-aged BP status.

Large research data suggest that the prevalence, awareness, treatment, and control of hypertension might be deteriorating, and the insouciance might be greater in middle-aged adults facing fewer short-term risks (27–29). Our findings emphasized the need to prevent and control an hypertension at the middle and old age. Emphasizing the long-term adverse effects of midlife hypertension might serve to motivate middle-aged adults to become aware of and address their elevated BP levels.

The strengths of this investigation are worth note. Strengths include the use of a large community-based cohort with the long duration of follow up, and the adequate events to test our hypotheses. Importantly, mid- and late-life BP patterns, rather than measured single BP value, were used to examine the effect on the risks of death and CHD.

Some limitations should be taken into consideration. First, although the impact of various confounding factors had been adjusted in our risk estimation models, we could not exclude residual measured or unmeasured confounders. And no statistical adjustment was performed based on the severity of medical conditions at Visit 4. However, the robustness of results was supported by the consistency of results in several sensitivity analyses. The analysis of E-value supported that the unmeasured confounders are unlikely to eliminate the observed association between BP patterns and study end points. Second, although all BP measurements were taken by trained staff according to standardized ARIC protocols and repeatability of measurements was high, some degree of measurement error was unavoidable for the adjudication of BP status (hypertension; normotension) based on the single visit only. Thus, misclassification that normal people might be misdiagnosed as hypertension was inevitable. However, such misclassification would likely attenuate findings toward the null and underestimates the effect of the observed associations. Third, some CHD events were found through hospital discharge codes, so someone who managed in an outpatient setting or not required hospital admission were unable to be identified. Fourth, as in all observational studies, we are unable to attribute causality to our observations. Thus, the interpretation of the findings should be cautious. Fifth, the findings may lack generalizability to all regions and other racial and ethnic groups (e.g., Asian and Hispanic).

In conclusion, the current findings underscore that the elderly hypertensive patient with midlife hypertension may be a distinctive individual at higher risk of death and CHD. The management of elderly hypertensive patients should not merely focus on the current BP status, but the middle-aged BP status. Furthermore, to optimize clinical and public health strategies toward minimizing the burden of CVD, it may be necessary to prevent, diagnose, and manage the hypertension throughout middle age. The primary prevention of hypertension through non-pharmacological measures throughout middle age, and the early detection and treatment of hypertension in older adults, promises to yield sustained benefits in the form of lower CVD risks later in life.

## Data Availability Statement

The data analyzed in this study was obtained from the ARIC Study, the following licenses/restrictions apply: human subjects' restrictions. Requests to access these datasets should be directed to the ARIC Study Coordinating Center.

## Ethics Statement

The studies involving human participants were reviewed and approved by The institutional review boards at all participating institutions approved the ARIC study protocol. The patients/participants provided their written informed consent to participate in this study.

## Author Contributions

ML and XZhu had full access to all the data in the study and take responsibility for the integrity of the data and the accuracy of the data analysis. ML, SZ, XZhu, and XL conceptualized and designed the study. XZho, XC, ZX, DY, YLin, YH, YLi, and LW acquired, analyzed, and interpreted the data. ML and SZ drafted the manuscript. SZ, XZho, XC, and LW performed the statistical analysis. XL obtained the funding. LW, XZhu, and XL were in-charge of the administrative, technical, or material support. XZhu and XL supervised the study. All authors critically revised the manuscript for important intellectual content.

## Conflict of Interest

The authors declare that the research was conducted in the absence of any commercial or financial relationships that could be construed as a potential conflict of interest.
