# Supplementary material for: Association of Mid- to Late-Life Blood Pressure Patterns With Risk of Subsequent Coronary Heart Disease and Death
Source: Front Cardiovasc Med. 2021 Feb 15;8:632514. doi: 10.3389/fcvm.2021.632514 (PMC7917074; doi:10.3389/fcvm.2021.632514)
Supplement: Supplementary file 1 [file Data_Sheet_1.PDF]

Supplementary Material

1 Supplementary Methods: The explanation for E-Value.

According to VanderWeele and Ding [VanderWeele TJ and Ding P (2017). ANN INTERN MED 167: 268-274.], the E-value (expressed on the risk ratio [RR] scale) is used to assess the robustness of exposure-outcome association to potential unmeasured or uncontrolled confounding. And it is defined as the minimum strength of association, that an unmeasured confounder would need to have with both the exposure and the outcome on the basis of the measured covariates, to fully explain away a specific exposure-outcome association. A small E-value implies that little unmeasured confounding would be demanded as an explanatory factor accounting for the effect estimate. A large E-value implies that considerable unmeasured confounding would be needed to explain away an effect estimate. Our focus has been on the E-values of the HR estimates involved in the all-cause mortality and coronary heart disease (CHD) events (Supplementary Table 7).

Our study revealed that the participants with the pattern of M-to-L HT with PC were associated with greater risk for all-cause mortality in comparison to the M-to-L HT group (HR, 1.49; 95% CI, 1.30-1.71). Based on the calculated E-value for the all-cause mortality, the observed HR of 1.49 could be accounted for by an unmeasured confounder that was associated with both the exposure and the outcome by a RR of 1.96-fold each, above and beyond the measured confounders, but weaker confounding could not do so.

Put simply, the calculated E-value of 1.96 would represent that if an existing unmeasured covariate came with a relative risk association with both the all-cause mortality and the pattern of M-to-L HT with PC by a RR of 1.96 at lowest, then the residual confounding could explain the observed association. HR was calculated directing at 5 well-known risk factors associated with the study endpoints (gender, BMI, TC, smoking status and previous diabetes history) (Supplementary Table 7). The result of E-value significantly higher than HR value would suggest that there was unlikely to exist an unmeasured or unknown confounder with substantially greater influence on the endpoint than those well-established cardiovascular risk factors.

Therefore, examining the E-values for all-cause mortality and CHD events and comparing with the HR estimates of known cardiovascular risk factors for these endpoints indicated it would be highly unlikely that an unmeasured confounder exists that could account for the association between mid- and late-life BP patterns and study endpoints (Supplementary Table 7).

2 Supplementary Figures and Tables

2.1 Supplementary Figures

Subgroup	Comparison groups	Hazard Ratio (95%CI)	*P* Value	*P* for interaction
**Age, y**				
< 65	M-NT and L-HT with EC	1.23 (0.93, 1.62)	0.147	0.138
	M-NT and L-HT with PC	1.39 (1.13, 1.71)	0.002	0.186
	M-to-L HT with EC	1.33 (1.07, 1.65)	0.011	0.138
	M-to-L HT with PC	2.12 (1.71, 2.64)	< 0.001	< 0.001
⩾65	M-NT and L-HT with EC	1.00 (0.79, 1.25)	0.967	0.138
	M-NT and L-HT with PC	1.19 (1.02, 1.39)	0.032	0.186
	M-to-L HT with EC	1.17 (0.98, 1.41)	0.082	0.138
	M-to-L HT with PC	1.31 (1.11, 1.56)	0.002	< 0.001
**Sex**				
Male	M-NT and L-HT with EC	1.07 (0.84, 1.35)	0.584	0.702
	M-NT and L-HT with PC	1.25 (1.05, 1.48)	0.012	0.822
	M-to-L HT with EC	1.14 (0.94, 1.39)	0.182	0.587
	M-to-L HT with PC	1.37 (1.13, 1.65)	0.001	0.544
Femele	M-NT and L-HT with EC	1.26 (0.97, 1.65)	0.089	0.702
	M-NT and L-HT with PC	1.29 (1.07, 1.54)	0.008	0.822
	M-to-L HT with EC	1.36 (1.11, 1.66)	0.003	0.587
	M-to-L HT with PC	1.63 (1.34, 1.99)	< 0.001	0.544
**Race**				
White	M-NT and L-HT with EC	1.17 (0.96, 1.43)	0.131	0.177
	M-NT and L-HT with PC	1.29 (1.12, 1.48)	< 0.001	0.086
	M-to-L HT with EC	1.19 (1.02, 1.40)	0.033	0.837
	M-to-L HT with PC	1.43 (1.22, 1.68)	< 0.001	0.684
Black	M-NT and L-HT with EC	1.05 (0.72, 1.54)	0.801	0.177
	M-NT and L-HT with PC	1.06 (0.78, 1.45)	0.721	0.086
	M-to-L HT with EC	1.30 (0.98, 1.73)	0.069	0.837
	M-to-L HT with PC	1.59 (1.21, 2.09)	0.001	0.684
**BMI, kg/m^2**				
< 30	M-NT and L-HT with EC	1.22 (0.98, 1.53)	0.077	0.620
	M-NT and L-HT with PC	1.27 (1.10, 1.48)	0.002	0.929
	M-to-L HT with EC	1.27 (1.07, 1.51)	0.007	0.688
	M-to-L HT with PC	1.43 (1.21, 1.69)	< 0.001	0.313
⩾30	M-NT and L-HT with EC	1.00 (0.74, 1.35)	0.998	0.620
	M-NT and L-HT with PC	1.21 (0.96, 1.53)	0.112	0.929
	M-to-L HT with EC	1.14 (0.90, 1.46)	0.281	0.688
	M-to-L HT with PC	1.55 (1.22, 1.96)	< 0.001	0.313
**LDL-C, mmol/L**				
< 3.4	M-NT and L-HT with EC	1.28 (1.03, 1.60)	0.027	0.849
	M-NT and L-HT with PC	1.35 (1.15, 1.58)	< 0.001	0.681
	M-to-L HT with EC	1.38 (1.16, 1.64)	< 0.001	0.533
	M-to-L HT with PC	1.61 (1.36, 1.91)	< 0.001	0.995
⩾3.4	M-NT and L-HT with EC	0.95 (0.71, 1.28)	0.743	0.849
	M-NT and L-HT with PC	1.15 (0.94, 1.42)	0.181	0.681
	M-to-L HT with EC	1.05 (0.83, 1.32)	0.714	0.533
	M-to-L HT with PC	1.35 (1.08, 1.69)	0.008	0.995
**History of DM**				
Yes	M-NT and L-HT with EC	1.15 (0.78, 1.69)	0.473	0.703
	M-NT and L-HT with PC	1.39 (1.02, 1.89)	0.037	0.510
	M-to-L HT with EC	1.26 (0.92, 1.73)	0.148	0.682
	M-to-L HT with PC	1.78 (1.31, 2.43)	< 0.001	0.156
No	M-NT and L-HT with EC	1.16 (0.95, 1.42)	0.156	0.703
	M-NT and L-HT with PC	1.24 (1.08, 1.43)	0.002	0.510
	M-to-L HT with EC	1.26 (1.08, 1.48)	0.004	0.682
	M-to-L HT with PC	1.43 (1.23, 1.67)	< 0.001	0.156

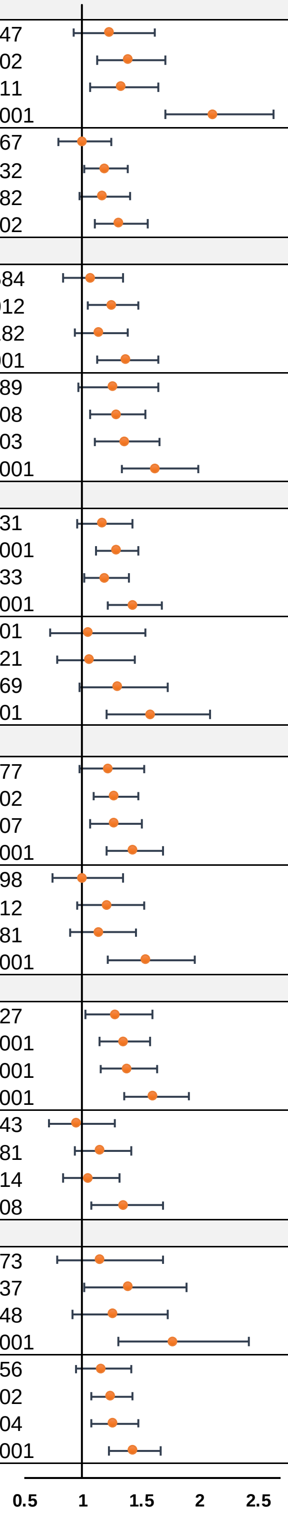

Supplementary Figure 1. Association of mid- to late-life BP patterns with all-cause mortality in key subgroups in the adjusted Cox models.

The Cox models adjusted for age, sex, race, education level, smoking status, drinking status, BMI, total cholesterol, HDL-C, LDL-C, eGFR, prevalent diabetes mellitus, coronary heart disease, stroke and heart failure, use of aspirin and statin, and use of antihypertensive drugs at Visit 4.

Adjusted hazard ratios were obtained after individually removing the original variable from the adjusted Cox model and replacing it with the dichotomous sub-group variable as well as its interaction with the groups of mid- to late-life BP patterns.

Subgroup	Comparison groups	Hazard Ratio (95%CI)	*P* Value	*P* for interaction
Age, y				
< 65	M-NT and L-HT with EC	1.48 (0.96, 2.28)	0.073	0.879
	M-NT and L-HT with PC	1.55 (1.11, 2.16)	0.009	0.636
	M-to-L HT with EC	1.71 (1.22, 2.41)	0.002	0.358
	M-to-L HT with PC	2.21 (1.55, 3.14)	< 0.001	0.524
⩾65	M-NT and L-HT with EC	1.21 (0.79, 1.85)	0.377	0.879
	M-NT and L-HT with PC	1.53 (1.12, 2.08)	0.008	0.636
	M-to-L HT with EC	1.61 (1.15, 2.25)	0.006	0.358
	M-to-L HT with PC	1.71 (1.23, 2.36)	0.001	0.524
Sex				
Male	M-NT and L-HT with EC	1.25 (0.86, 1.84)	0.244	0.605
	M-NT and L-HT with PC	1.30 (0.97, 1.75)	0.083	0.047
	M-to-L HT with EC	1.56 (1.14, 2.13)	0.005	0.502
	M-to-L HT with PC	1.58 (1.16, 2.17)	0.004	0.018
Femele	M-NT and L-HT with EC	1.54 (0.94, 2.54)	0.090	0.605
	M-NT and L-HT with PC	2.02 (1.43, 2.85)	< 0.001	0.047
	M-to-L HT with EC	1.85 (1.27, 2.69)	0.001	0.502
	M-to-L HT with PC	2.47 (1.71, 3.58)	< 0.001	0.018
Race				
White	M-NT and L-HT with EC	1.61 (1.16, 2.24)	0.005	0.149
	M-NT and L-HT with PC	1.66 (1.30, 2.11)	< 0.001	0.559
	M-to-L HT with EC	1.77 (1.35, 2.31)	< 0.001	0.965
	M-to-L HT with PC	2.08 (1.59, 2.72)	< 0.001	0.589
Black	M-NT and L-HT with EC	0.62 (0.30, 1.32)	0.217	0.149
	M-NT and L-HT with PC	1.10 (0.61, 1.97)	0.747	0.559
	M-to-L HT with EC	1.13 (0.65, 1.99)	0.664	0.965
	M-to-L HT with PC	1.17 (0.67, 2.05)	0.584	0.589
BMI, kg/m^2				
< 30	M-NT and L-HT with EC	1.46 (1.00, 2.13)	0.050	0.573
	M-NT and L-HT with PC	1.53 (1.16, 2.01)	0.002	0.582
	M-to-L HT with EC	1.80 (1.34, 2.42)	< 0.001	0.378
	M-to-L HT with PC	1.93 (1.43, 2.59)	< 0.001	0.936
⩾30	M-NT and L-HT with EC	1.10 (0.66, 1.81)	0.724	0.573
	M-NT and L-HT with PC	1.53 (1.03, 2.28)	0.036	0.582
	M-to-L HT with EC	1.38 (0.91, 2.09)	0.130	0.378
	M-to-L HT with PC	1.69 (1.13, 2.54)	0.011	0.936
LDL-C, mmol/L				
< 3.4	M-NT and L-HT with EC	1.57 (1.06, 2.32)	0.024	0.337
	M-NT and L-HT with PC	1.40 (1.03, 1.91)	0.032	0.317
	M-to-L HT with EC	1.82 (1.32, 2.49)	< 0.001	0.371
	M-to-L HT with PC	2.17 (1.58, 2.97)	< 0.001	0.054
⩾3.4	M-NT and L-HT with EC	1.10 (0.69, 1.77)	0.682	0.337
	M-NT and L-HT with PC	1.72 (1.24, 2.38)	0.001	0.317
	M-to-L HT with EC	1.46 (1.01, 2.11)	0.045	0.371
	M-to-L HT with PC	1.55 (1.07, 2.24)	0.019	0.054
History of DM				
Yes	M-NT and L-HT with EC	1.04 (0.55, 1.96)	0.904	0.829
	M-NT and L-HT with PC	1.31 (0.77, 2.23)	0.326	0.797
	M-to-L HT with EC	1.54 (0.91, 2.60)	0.110	0.484
	M-to-L HT with PC	1.84 (1.09, 3.09)	0.022	0.490
No	M-NT and L-HT with EC	1.46 (1.03, 2.06)	0.034	0.829
	M-NT and L-HT with PC	1.61 (1.26, 2.06)	< 0.001	0.797
	M-to-L HT with EC	1.66 (1.26, 2.19)	< 0.001	0.484
	M-to-L HT with PC	1.85 (1.41, 2.44)	< 0.001	0.490

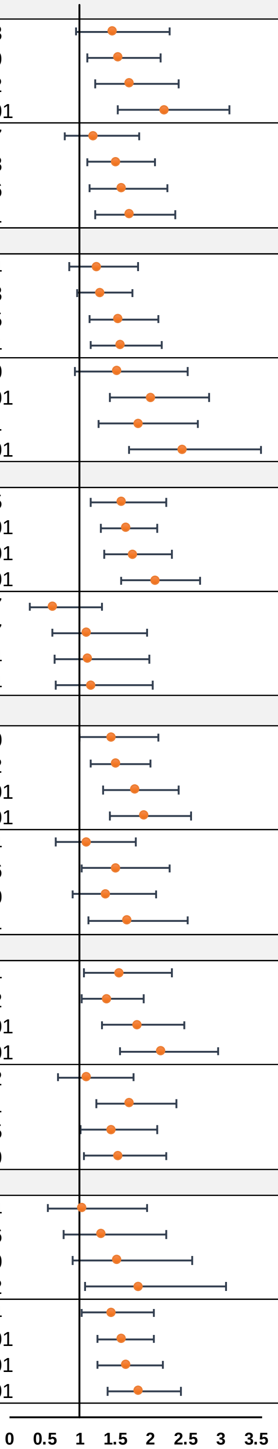

Supplementary Figure 2. Association of mid- to late-life BP patterns with coronary heart disease in key subgroups in the adjusted Cox models.

The Cox models adjusted for age, sex, race, education level, smoking status, drinking status, BMI, total cholesterol, HDL-C, LDL-C, eGFR, prevalent diabetes mellitus, coronary heart disease, stroke and heart failure, use of aspirin and statin, and use of antihypertensive drugs at Visit 4.

Adjusted hazard ratios were obtained after individually removing the original variable from the adjusted Cox model and replacing it with the dichotomous sub-group variable as well as its interaction with the groups of mid- to late-life BP patterns.

2.2 Supplementary Tables

Supplementary Table 1. Association between covariates at visit 4 and all-cause mortality by univariate analysis.

Variable at visit 4	HR (95% CI)	*P* Value
Age (Per 1 year)	1.11 (1.11, 1.12)	< 0.001
Sex (Man vs Woman)	1.62 (1.51, 1.74)	< 0.001
Race (White vs Black)	0.79 (0.73, 0.86)	< 0.001
Education level (Advanced vs Basic or 0 y)	0.55 (0.50, 0.60)	< 0.001
Smoking status (Current vs Never)	1.40 (1.29, 1.51)	< 0.001
Drinking status (Current vs Never)	1.23 (1.12, 1.36)	< 0.001
BMI (Per 1 kg/m^2)	1.01 (1.00, 1.01)	0.090
Total cholesterol (Per 1 mmol/L)	0.88 (0.85, 0.92)	< 0.001
HDL-C (Per 1 mmol/L)	0.74 (0.68, 0.81)	< 0.001
LDL-C (Per 1 mmol/L)	0.91 (0.88, 0.95)	< 0.001
eGFR (Per 1 mL/min/1.73 m^2)	0.98 (0.99, 0.99)	< 0.001
Prevalent diabetes mellitus (Yes vs No)	2.04 (1.88, 2.21)	< 0.001
Prevalent coronary heart disease (Yes vs No)	2.48 (2.19, 2.81)	< 0.001
Prevalent stroke (Yes vs No)	2.89 (2.44, 3.42)	< 0.001
Prevalent heart failure (Yes vs No)	2.43 (2.14, 2.75)	< 0.001
Use of aspirin (Yes vs No)	1.18 (1.10, 1.27)	< 0.001
Use of statin (Yes vs No)	1.32 (1.19, 1.46)	< 0.001
SBP (Per 1 mmHg)	1.01 (1.01, 1.02)	< 0.001
DBP (Per 1 mmHg)	0.99 (0.99, 1.00)	< 0.001
Use of antihypertensive drugs (Yes vs No)	1.67 (1.56, 1.79)	< 0.001

BMI: body mass index; HDL-C: high-density lipoprotein cholesterol; LDL-C: low-density lipoprotein cholesterol; eGFR: estimated glomerular filtration rate; SBP: systolic blood pressure; DBP: Diastolic blood pressure.

Supplementary Table 2. Participant characteristics at visit 4 in midlife normotension and hypertension group.

Characteristic, n (%) or mean (SD)	Midlife hypertension	Midlife normotension	*P* Value
No.	3371	6458	
Age, y	64.0 ± 5.6	62.2 ± 5.6	< 0.001
Sex, No. (%)			< 0.001
Men	1373 (40.7)	2915 (45.1)	
Women	1998 (59.3)	3543 (54.9)	
Race, No. (%)			< 0.001
Black	1077 (31.9)	926 (14.3)	
White	2294 (68.1)	5532 (85.7)	
BMI, kg/m^2	30.3 ± 6.1	27.9 ± 5.1	< 0.001
Systolic BP, mm Hg	136.0 ± 20.1	122.8 ± 16.4	< 0.001
Diastolic BP, mm Hg	73.3 ± 11.2	69.6 ± 9.5	< 0.001
Total cholesterol, mmol/L	5.2 ± 1.0	5.2 ± 0.9	0.027
HDL-C, mmol/L	1.3 ± 0.4	1.3 ± 0.4	< 0.001
LDL-C, mmol/L	3.1 ± 0.9	3.2 ± 0.9	0.003
eGFR, mL/min/1.73 m^2	84.0 ± 18.5	87.2 ± 14.2	< 0.001
History of diabetes mellitus, No. (%)	826 (24.5)	712 (11.0)	< 0.001
History of CHD, No. (%)	278 (8.2)	198 (3.1)	< 0.001
History of stroke, No. (%)	148 (4.4)	68 (1.1)	< 0.001
History of heart failure, No. (%)	407 (12.1)	56 (0.9)	< 0.001
Education level, No. (%)			< 0.001
Basic or 0 y	826 (24.5)	957 (14.8)	
Intermediate	1389 (41.2)	2780 (43.0)	
Advanced	1156 (34.3)	2721 (42.1)	
Smoking, No. (%)			< 0.001
Current smoker	424 (12.6)	1035 (16.0)	
Former smoker	1457 (43.2)	2806 (43.4)	
Never smoker	1490 (44.2)	2617 (40.5)	
Drinking, No. (%)			< 0.001
Current drinker	1414 (41.9)	3547 (54.9)	
Former drinker	1106 (32.8)	1770 (27.4)	
Never drinker	851 (25.2)	1141 (17.7)	
Aspirin, No. (%)	3440 (53.3)	2059 (61.1)	< 0.001
Statin, No. (%)	549 (8.5)	487 (14.4)	< 0.001
Antihypertensive, No. (%)	2734 (81.1)	1355 (21.0)	< 0.001
Characteristics at midlife (Visit 1)			
Age, y	55.1 ± 5.7	53.3 ± 5.6	< 0.001
Systolic BP, mm Hg	130.3 ± 19.6	113.3 ± 12.1	< 0.001
Diastolic BP, mm Hg	78.5 ± 11.6	70.0 ± 8.5	< 0.001

Data presented as mean (SD) or percentage. BP: blood pressure. BMI: body mass index; HDL-C: high-density lipoprotein cholesterol; LDL-C: low-density lipoprotein cholesterol; eGFR: estimated glomerular filtration rate; CHD: coronary heart disease.

Supplementary Table 3. Hazard ratios from the Cox models for all-cause mortality and CHD for midlife hypertension group vs midlife normotension group (Reference group).

Outcome	No./Total No. (%)	Model 1		Model 2		Model 3	
		Hazard Ratio (95% CI)	*P* Value	Hazard Ratio (95% CI)	*P* Value	Hazard Ratio (95% CI)	*P* Value
All-cause mortality							
Midlife normotension	1720/6458 (26.6)	1.46 (1.36-1.57)	< 0.001	1.23 (1.13-1.33)	< 0.001	1.14 (1.04-1.25)	0.006
Midlife hypertension	1414/3371 (41.9)						
Coronary heart disease							
Midlife normotension	532/6458 (8.2)	1.94 (1.71-2.20)	< 0.001	1.48 (1.30-1.70)	< 0.001	1.28 (1.10-1.50)	0.002
Midlife hypertension	528/3371 (15.7)						

Model 1: adjusted for age, sex, race at Visit 4;
Model 2: adjusted for model 1 + education level, smoking status, drinking status, BMI, total cholesterol, HDL-C, LDL-C, eGFR, prevalent diabetes mellitus, CHD, stroke and heart failure, use of aspirin and statin at Visit 4;
Model 3: adjusted for model 2 + SBP, DBP, use of antihypertensive drugs at Visit 4.
BMI: body mass index; HDL-C: high-density lipoprotein cholesterol; LDL-C: low-density lipoprotein cholesterol; eGFR: estimated glomerular filtration rate; CHD: coronary heart disease; SBP: systolic blood pressure; DBP: diastolic blood pressure.

Supplementary Table 4. Hazard ratios from the Cox models for all-cause mortality and CHD for midlife hypertension group vs midlife normotension group (Reference group) in participants without CHD, MI, stroke or HF.

Outcome	No./Total No. (%)	Model 1		Model 2		Model 3	
		Hazard Ratio (95% CI)	*P* Value	Hazard Ratio (95% CI)	*P* Value	Hazard Ratio (95% CI)	*P* Value
All-cause mortality							
Midlife normotension	1560/6154 (25.3)	1.33 (1.22-1.44)	< 0.001	1.26 (1.16-1.37)	< 0.001	1.19 (1.07-1.32)	0.001
Midlife hypertension	1000/2652 (37.7)						
Coronary heart disease							
Midlife normotension	456/6154 (7.4)	1.68 (1.45-1.94)	< 0.001	1.48 (1.27-1.72)	< 0.001	1.25 (1.04-1.50)	0.017
Midlife hypertension	337/2652 (12.7)						

Model 1: adjusted for age, sex, race at Visit 4;
Model 2: adjusted for model 1 + education level, smoking status, drinking status, BMI, total cholesterol, HDL-C, LDL-C, eGFR, prevalent diabetes mellitus, use of aspirin and statin at Visit 4;
Model 3: adjusted for model 2 + SBP, DBP, use of antihypertensive drugs at Visit 4.
BMI: body mass index; HDL-C: high-density lipoprotein cholesterol; LDL-C: low-density lipoprotein cholesterol; eGFR: estimated glomerular filtration rate; CHD: coronary heart disease; MI: myocardial infarction; HF: heart failure; SBP: systolic blood pressure; DBP: diastolic blood pressure.

Supplementary Table 5. Hazard ratios from the Cox models for all-cause mortality and CHD for midlife hypertension group vs midlife normotension group (Reference group) in participants without hypotension at visit 1 and 4.

Outcome	No./Total No. (%)	Model 1		Model 2		Model 3	
		Hazard Ratio (95% CI)	*P* Value	Hazard Ratio (95% CI)	*P* Value	Hazard Ratio (95% CI)	*P* Value
All-cause mortality							
Midlife normotension	1314/5179 (25.4)	1.50 (1.39-1.63)	< 0.001	1.28 (1.17-1.40)	< 0.001	1.18 (1.07-1.31)	0.002
Midlife hypertension	1201/2936 (40.9)						
Coronary heart disease							
Midlife normotension	428/5179 (8.3)	1.85 (1.61-2.13)	< 0.001	1.45 (1.25-1.68)	< 0.001	1.24 (1.04-1.47)	0.016
Midlife hypertension	435/2936 (14.8)						

Model 1: adjusted for age, sex, race at Visit 4;
Model 2: adjusted for model 1 + education level, smoking status, drinking status, BMI, total cholesterol, HDL-C, LDL-C, eGFR, prevalent diabetes mellitus, CHD, stroke and heart failure, use of aspirin and statin at Visit 4;
Model 3: adjusted for model 2 + SBP, DBP, use of antihypertensive drugs at Visit 4.
BMI: body mass index; HDL-C: high-density lipoprotein cholesterol; LDL-C: low-density lipoprotein cholesterol; eGFR: estimated glomerular filtration rate; CHD: coronary heart disease; SBP: systolic blood pressure; DBP: diastolic blood pressure.

Supplementary Table 6. Hazard ratios from the Cox models for all-cause mortality and CHD among mid- to late-life BP models in participants without CHD, MI, stroke or HF.

Outcome	No./Total No. (%)	Model 1		Model 2		Model 3	
		HR (95% CI)	*P* Value	HR (95% CI)	*P* Value	HR (95% CI)	*P* Value
All-cause mortality							
M-to-L NT	982/4288 (22.9)	1.00 (Reference)	—	1.00 (Reference)	—	1.00 (Reference)	—
L-HT with EC							
M-NT and L-HT with EC	211/788 (26.8)	1.12 (0.97-1.30)	0.133	1.04 (0.90-1.21)	0.588	1.11 (0.91-1.35)	0.303
M-to-L HT with EC	444/1377 (32.2)	1.32 (1.18-1.48)	< 0.001	1.25 (1.11-1.41)	< 0.001	1.31 (1.13-1.53)	0.001
L-HT with PC							
M-NT and L-HT with PC	367/1078 (34.0)	1.33 (1.18-1.50)	< 0.001	1.26 (1.12-1.43)	< 0.001	1.29 (1.13-1.47)	< 0.001
M-to-L HT with PC	556/1275 (43.6)	1.55 (1.39-1.73)	< 0.001	1.43 (1.28-1.60)	< 0.001	1.50 (1.29-1.74)	< 0.001
Coronary heart disease							
M-to-L NT	264/4288 (6.2)	1.00 (Reference)	—	1.00 (Reference)	—	1.00 (Reference)	—
L-HT with EC							
M-NT and L-HT with EC	70/788 (8.9)	1.41 (1.09-1.84)	0.010	1.24 (0.95-1.62)	0.120	1.26 (0.90-1.78)	0.185
M-to-L HT with EC	158/1377 (11.5)	1.88 (1.54-2.30)	< 0.001	1.66 (1.35-2.05)	< 0.001	1.69 (1.29-2.22)	< 0.001
L-HT with PC							
M-NT and L-HT with PC	122/1078 (11.3)	1.82 (1.47-2.26)	< 0.001	1.67 (1.34-2.08)	< 0.001	1.69 (1.33-2.13)	< 0.001
M-to-L HT with PC	179/1275 (14.0)	2.20 (1.80-2.68)	< 0.001	1.84 (1.50-2.26)	< 0.001	1.87 (1.43-2.44)	< 0.001

Model 1: adjusted for age, sex, race at Visit 4;
Model 2: adjusted for model 1 + education level, smoking status, drinking status, BMI, total cholesterol, HDL-C, LDL-C, eGFR, prevalent diabetes mellitus, use of aspirin and statin at Visit 4;
Model 3: adjusted for model 2 + use of antihypertensive drugs at Visit 4.
M-to-L NT: mid- to late-life normotension; M-NT and L-HT with EC: midlife normotension and late-life hypertension with effective control; M-to-L HT with EC: mid- to late-life hypertension with effective control; M-NT and L-HT with PC: midlife normotension and late-life hypertension with poor control; M-to-L HT with PC: mid- to late-life hypertension with poor control; BP: blood pressure; BMI: body mass index; HDL-C: high-density lipoprotein cholesterol; LDL-C: low-density lipoprotein cholesterol; eGFR: estimated glomerular filtration rate; CHD: coronary heart disease; MI: myocardial infarction; HF: heart failure.

Supplementary Table 7. Hazard ratios from the Cox models for all-cause mortality and CHD among mid- to late-life BP models in participants without hypotension at visit 1 and 4.

Outcome	No./Total No. (%)	Model 1		Model 2		Model 3	
		HR (95% CI)	*P* Value	HR (95% CI)	*P* Value	HR (95% CI)	*P* Value
**All-cause mortality**							
M-to-L NT	762/3411 (22.3)	1.00 (Reference)	—	1.00 (Reference)	—	1.00 (Reference)	—
L-HT with EC							
M-NT and L-HT with EC	248/840 (29.5)	1.28 (1.11-1.47)	0.001	1.12 (0.97-1.30)	0.121	1.20 (0.99-1.45)	0.064
M-to-L HT with EC	577/1632 (35.4)	1.52 (1.36-1.70)	< 0.001	1.25 (1.11-1.40)	< 0.001	1.32 (1.13-1.54)	< 0.001
L-HT with PC							
M-NT and L-HT with PC	304/928 (32.8)	1.34 (1.17-1.53)	< 0.001	1.26 (1.10-1.44)	0.001	1.28 (1.12-1.48)	< 0.001
M-to-L HT with PC	624/1304 (47.9)	1.87 (1.67-2.09)	< 0.001	1.54 (1.37-1.73)	< 0.001	1.62 (1.40-1.88)	< 0.001
**Coronary heart disease**							
M-to-L NT	222/3411 (6.5)	1.00 (Reference)	—	1.00 (Reference)	—	1.00 (Reference)	—
L-HT with EC							
M-NT and L-HT with EC	93/840 (11.1)	1.72 (1.35-2.19)	< 0.001	1.33 (1.04-1.70)	0.025	1.28 (0.92-1.77)	0.138
M-to-L HT with EC	229/1632 (14.0)	2.30 (1.91-2.77)	< 0.001	1.72 (1.41-2.09)	< 0.001	1.66 (1.28-2.17)	< 0.001
L-HT with PC							
M-NT and L-HT with PC	113/928 (12.2)	1.92 (1.53-2.41)	< 0.001	1.72 (1.36-2.16)	< 0.001	1.69 (1.33-2.15)	< 0.001
M-to-L HT with PC	206/1304 (15.8)	2.52 (2.07-3.07)	< 0.001	1.83 (1.49-2.26)	< 0.001	1.78 (1.37-2.32)	< 0.001

Model 1: adjusted for age, sex, race at Visit 4;
Model 2: adjusted for model 1 + education level, smoking status, drinking status, BMI, total cholesterol, HDL-C, LDL-C, eGFR, prevalent diabetes mellitus, coronary heart disease, stroke and heart failure, use of aspirin and statin at Visit 4;
Model 3: adjusted for model 2 + use of antihypertensive drugs at Visit 4.
M-to-L NT: mid- to late-life normotension; M-NT and L-HT with EC: midlife normotension and late-life hypertension with effective control; M-to-L HT with EC: mid- to late-life hypertension with effective control; M-NT and L-HT with PC: midlife normotension and late-life hypertension with poor control; M-to-L HT with PC: mid- to late-life hypertension with poor control; BP: blood pressure; BMI: body mass index; HDL-C: high-density lipoprotein cholesterol; LDL-C: low-density lipoprotein cholesterol; eGFR: estimated glomerular filtration rate; CHD: coronary heart disease.

Supplementary Table 8. Hazard ratios from the Cox models for all-cause mortality and coronary heart disease among mid- to late-life BP models in participants with the age < 60 years in visit 1 (Mid-life) and the age ⩾ 60 years in visit 4 (Late-life).

Outcome	No./Total No. (%)	Model 1		Model 2		Model 3	
		HR (95% CI)	*P* Value	HR (95% CI)	*P* Value	HR (95% CI)	*P* Value
All-cause mortality							
M-to-L NT	481/1925 (25.0)	1.00 (Reference)	—	1.00 (Reference)	—	1.00 (Reference)	—
L-HT with EC							
M-NT and L-HT with EC	116/408 (28.4)	1.08 (0.88-1.33)	0.452	1.00 (0.81-1.23)	0.963	0.99 (0.76-1.29)	0.930
M-to-L HT with EC	282/812 (34.7)	1.43 (1.23-1.66)	< 0.001	1.21 (1.03-1.42)	0.018	1.21 (0.98-1.48)	0.076
L-HT with PC							
M-NT and L-HT with PC	189 /547 (34.6)	1.39 (1.17-1.64)	< 0.001	1.31 (1.10-1.55)	0.002	1.30 (1.08-1.56)	0.005
M-to-L HT with PC	333/752 (44.3)	1.88 (1.62-2.17)	< 0.001	1.57 (1.34-1.83)	< 0.001	1.56 (1.27-1.91)	< 0.001
Coronary heart disease							
M-to-L NT	141/1925 (7.3)	1.00 (Reference)	—	1.00 (Reference)	—	1.00 (Reference)	—
L-HT with EC							
M-NT and L-HT with EC	47/408 (11.5)	1.55 (1.12-2.16)	0.009	1.23 (0.87-1.72)	0.243	1.38 (0.90-2.12)	0.137
M-to-L HT with EC	113/812 (13.9)	2.09 (1.62-2.68)	< 0.001	1.62 (1.25-2.11)	< 0.001	1.80 (1.28-2.51)	0.001
L-HT with PC							
M-NT and L-HT with PC	72/547 (13.2)	1.92 (1.44-2.55)	< 0.001	1.65 (1.23-2.20)	0.001	1.74 (1.27-2.37)	< 0.001
M-to-L HT with PC	129/752 (17.2)	2.78 (2.17-3.56)	< 0.001	2.03 (1.57-2.64)	< 0.001	2.24 (1.61-3.13)	< 0.001

Model 1: adjusted for age, sex, race at Visit 4;
Model 2: adjusted for model 1 + education level, smoking status, drinking status, BMI, total cholesterol, HDL-C, LDL-C, eGFR, prevalent diabetes mellitus, coronary heart disease, stroke and heart failure, use of aspirin and statin at Visit 4;
Model 3: adjusted for model 2 + use of antihypertensive drugs at Visit 4.
M-to-L NT: mid- to late-life normotension; M-NT and L-HT with EC: midlife normotension and late-life hypertension with effective control; M-to-L HT with EC: mid- to late-life hypertension with effective control; M-NT and L-HT with PC: midlife normotension and late-life hypertension with poor control; M-to-L HT with PC: mid- to late-life hypertension with poor control; BP: blood pressure; BMI: body mass index; HDL-C: high-density lipoprotein cholesterol; LDL-C: low-density lipoprotein cholesterol; eGFR: estimated glomerular filtration rate.

Supplementary Table 9. Hazard ratios from the Cox models for all-cause mortality and coronary heart disease among mid- to late-life BP models in a post hoc analysis when midlife hypertension was also classified into EC and PC groups.

Outcome	No./Total No. (%)	Model 1		Model 2		Model 3	
		HR (95% CI)	*P* Value	HR (95% CI)	*P* Value	HR (95% CI)	*P* Value
All-cause mortality							
M-to-L NT	1045/4415 (23.7)	1.00 (Reference)	—	1.00 (Reference)	—	1.00 (Reference)	—
L-HT with EC							
M-NT and L-HT with EC	264/882 (29.9)	1.22 (1.07-1.40)	0.004	1.10 (0.96-1.26)	0.193	1.15 (0.96-1.37)	0.124
M-HT with EC and L-HT with EC	365/1125 (32.4)	1.32 (1.17-1.49)	< 0.001	1.08 (0.96-1.23)	0.213	1.12 (0.96-1.31)	0.163
M-HT with PC and L-HT with EC	346/727 (47.6)	1.83 (1.61-2.07)	< 0.001	1.39 (1.22-1.58)	< 0.001	1.43 (1.22-1.69)	< 0.001
L-HT with PC							
M-NT and L-HT with PC	411/1161 (35.4)	1.35 (1.20-1.51)	< 0.001	1.24 (1.10-1.39)	< 0.001	1.27 (1.12-1.44)	< 0.001
M-HT with EC and L-HT with PC	253/616 (41.1)	1.65 (1.44-1.90)	< 0.001	1.40 (1.21-1.61)	< 0.001	1.44 (1.22-1.70)	< 0.001
M-HT with PC and L-HT with PC	450/903 (49.8)	1.75 (1.56-1.97)	< 0.001	1.48 (1.31-1.66)	< 0.001	1.52 (1.31-1.75)	< 0.001
Coronary heart disease							
M-to-L NT	292/4415 (6.6)	1.00 (Reference)	—	1.00 (Reference)	—	1.00 (Reference)	—
L-HT with EC							
M-NT and L-HT with EC	99/882 (11.2)	1.68 (1.34-2.11)	< 0.001	1.34 (1.06-1.69)	0.013	1.33 (0.99-1.80)	0.059
M-HT with EC and L-HT with EC	160/1125 (14.2)	2.28 (1.88-2.77)	< 0.001	1.73 (1.41-2.12)	< 0.001	1.72 (1.33-2.23)	< 0.001
M-HT with PC and L-HT with EC	129/727 (17.7)	2.80 (2.27-3.46)	< 0.001	1.84 (1.47-2.30)	< 0.001	1.83 (1.39-2.42)	< 0.001
L-HT with PC							
M-NT and L-HT with PC	141/1161 (12.1)	1.81 (1.48-2.22)	< 0.001	1.54 (1.25-1.89)	< 0.001	1.53 (1.23-1.92)	< 0.001
M-HT with EC and L-HT with PC	84/616 (13.6)	2.07 (1.62-2.65)	< 0.001	1.53 (1.19-1.97)	0.001	1.52 (1.14-2.04)	0.005
M-HT with PC and L-HT with PC	155/903 (17.2)	2.59 (2.12-3.17)	< 0.001	1.91 (1.55-2.36)	< 0.001	1.91 (1.48-2.46)	< 0.001

Model 1: adjusted for age, sex, race at Visit 4;
Model 2: adjusted for model 1 + education level, smoking status, drinking status, BMI, total cholesterol, HDL-C, LDL-C, eGFR, prevalent diabetes mellitus, coronary heart disease, stroke and heart failure, use of aspirin and statin at Visit 4;
Model 3: adjusted for model 2 + use of antihypertensive drugs at Visit 4.
M-to-L NT: mid- to late-life normotension; M-NT: midlife normotension; M-HT: midlife hypertension; L-NT: late-life normotension; L-HT: late-life hypertension; EC: effective BP control; PC: poor BP control; BP: blood pressure; BMI: body mass index; HDL-C: high-density lipoprotein cholesterol; LDL-C: low-density lipoprotein cholesterol; eGFR: estimated glomerular filtration rate.

Supplementary Table 10. E-Value for the effect of mid- and late-life BP patterns on all-cause mortality and coronary heart disease.

Outcome	Reference group	Comparison groups	E-value for HR estimate	Cardiovascular risk factors *
				Variable: Level, HR (95% CI) †
All-cause mortality	M-to-L NT	M-NT and L-HT with EC	—	Gender: Man vs. Women, 1.48 (1.29-1.60);<br>BMI: $\geq$ 30 kg/m^2 vs. < 30 kg/m^2, 1.03 (0.95-1.11);<br>TC: $\geq$ 5.2 mmol/L vs. < 5.2 mmol/L, 1.04 (0.94-1.16)<br>Smoking: Yes vs. No, 1.56 (1.44-1.69);<br>Diabetes: Yes vs. No, 1.66 (1.53-1.82).
		M-NT and L-HT with PC	1.64	
		M-to-L HT with EC	1.59	
		M-to-L HT with PC	1.96	
Coronary heart disease	M-to-L NT	M-NT and L-HT with EC	—	Gender: Man vs. Women, 1.56 (1.35-1.80);<br>BMI: $\geq$ 30 kg/m^2 vs. < 30 kg/m^2, 0.97 (0.85-1.11);<br>TC: $\geq$ 5.2 mmol/L vs. < 5.2 mmol/L, 1.02 (0.85-1.21)<br>Smoking: Yes vs. No, 1.60 (1.38-1.85);<br>Diabetes: Yes vs. No, 1.93 (1.67-2.22).
		M-NT and L-HT with PC	2.43	
		M-to-L HT with EC	2.69	
		M-to-L HT with PC	3.15	

M-to-L NT: mid- to late-life normotension; M-NT and L-HT with EC: midlife normotension and late-life hypertension with effective control; M-NT and L-HT with PC: midlife normotension and late-life hypertension with poor control; M-to-L HT with EC: mid- to late-life hypertension with effective control; M-to-L HT with PC: mid- to late-life hypertension with poor control; BP: blood pressure; HR: hazard ratios; CI: confidence intervals; BMI: body mass index; TC: total cholesterol. The detailed description of E-value in the above supplementary methods. * HR (95% CI) of known cardiovascular risk factors for each outcome are used to compare with E-value. † HR (95% CI) from the Cox models adjusted for age, sex, race, education level, smoking status, drinking status, BMI, total cholesterol, HDL-C, LDL-C, eGFR, prevalent diabetes mellitus, coronary heart disease, stroke and heart failure, use of aspirin and statin, and use of antihypertensive drugs at Visit 4, but the exploratory variable should be removed in accordance with specific conditions.
